# Changes in the Water-Energy Coupling Relationship in Grain Production: A Case Study of the North China Plain

**DOI:** 10.3390/ijerph19159527

**Published:** 2022-08-03

**Authors:** Xue Wang, Xiubin Li, Xingyuan Xiao, Limeng Fan, Lijun Zuo

**Affiliations:** 1 Key Laboratory of Land Surface Pattern and Simulation, Institute of Geographic Sciences and Natural Resources Research, Chinese Academy of Sciences, Beijing 100101, China; lixb@igsnrr.ac.cn; 2 College of Resources and Environment, University of Chinese Academy of Sciences, Beijing 100049, China; 3 College of Geomatics, Shandong University of Science and Technology, Qingdao 266590, China; skd991338@sdust.edu.cn; 4 Qingdao Hengxing University of Science and Technology, Qingdao 266100, China; lemon_gre@126.com; 5 Aerospace Information Research Institute, Chinese Academy of Sciences, Beijing 100101, China; zuolj@aircas.ac.cn

**Keywords:** water-energy relation, grain production, coupling index, FEW nexus, staple food

## Abstract

Water consumption and energy consumption are inevitable in grain production, but few studies have focused on the integrated assessment of these two indicators and their relationships. To address the research deficiency, taking the North China Plain (NCP) as a case study, this paper quantifies the changes in grain crop planting structure and the accompanying changes in irrigation water consumption (IWC) and energy consumption (EC) in the NCP. On this basis, the water-energy coupling index (CI) is constructed to analyze the water-energy coupling relationship in the context of grain crop planting structure change. The results revealed that the sown area of three of the four main grain crops in the NCP, namely winter wheat, summer maize, and rice, roughly increased in the south and decreased in the north, while the sown area of spring maize increased in most counties where it was planted in the NCP from 2000 to 2015. With the change of grain crop planting structure, IWC and EC of winter wheat in the NCP decreased by 19.87 × 10^6^ m^3^ and 16.78 × 10^8^ MJ, respectively, mainly distributed in the Beijing-Tianjin-Hebei region, while IWC and EC of other crops all increased. In terms of CI values, although that of spring maize increased, those of winter wheat, summer maize, and rice all decreased, and the overall CI values of grain production in the NCP decreased from 0.442 in 2000 to 0.438 in 2015, indicating that grain crop distribution has been optimized toward a less water- and energy-intensive and more sustainable layout in the NCP. This paper can add case and methodological support to the food-water-energy (FEW) nexus research and can also provide policy suggestions for regional crop optimization layout and conservation of both water and energy resources.

## 1. Introduction

Agricultural production is inseparable from water supply [1,2]. At the same time, agricultural production processes also require energy consumption [3]. Specifically, the sowing, irrigation, and harvesting of crops need to consume fuel and electrical energy; the production processes of agricultural inputs, such as fertilizer, pesticides, and plastic films, also require energy consumption. It is estimated that agriculture accounts for 70% of total global freshwater withdrawals and 30% of total global energy consumed [4]. In different countries or regions, or in different periods of the same region, there are often differences in agricultural planting structure and, consequently, in the amount of water and energy consumed [5,6,7].

Water consumption for agricultural production, as an important component of the food-water-energy (FEW) nexus, has been a hot issue in academic research [8]. Many scholars have focused on assessing water use for irrigation during agricultural production using models [9,10,11], while some scholars also invoke the concepts of water footprint, which integrates irrigation water, or blue water, as well as natural precipitation, or green water, and water used to dilute agricultural pollutants, or grey water, to comprehensively and systematically analyze the water footprints and the differences in their components at different spatial scales or different crop types [12,13,14,15,16]. At the same time, energy consumption for agricultural production has also attracted the attention of many scholars. Most of the relevant studies focused on the assessment of the amount of direct and indirect energy consumption in the production of crops, including wheat (*Triticum aestivum*), maize (*Zea mays*), and rice (*Oryza sativa*), using the life cycle assessment (LCA) approach [17,18,19,20]. However, to our knowledge, few studies have focused on the integrated assessment of water and energy consumption in agricultural production [21].

To address the research deficiency, taking the North China Plain (NCP) as a case study, this study quantifies the irrigation water consumption (IWC) and energy consumption (EC) of grain production in the NCP from 2000 to 2015 and estimates the changes in the water-energy coupling relationship in the context of agricultural planting structure change in the NCP. The NCP is well-known as the bread-basket of China, which supplies about 50% of wheat production and 33% of maize production [22]. However, it is also a globally representative area presenting a severe contradiction between groundwater exploitation and grain production [23,24]. Specifically, the irrigation water consumption of winter wheat is regarded as the main reason for the over-exploitation of groundwater and the decline of groundwater level [25,26]. However, in recent years, the agricultural planting structure in this region is changing, represented by the spatial shift of the sown area of winter wheat [27]. In this context, the IWC, together with EC and their coupling relationship in grain production in the NCP, may also change. Analyzing the change of the water-energy coupling relationship in grain production in the NCP can not only improve the understanding of the FEW nexus as a case study, but also provide scientific support for the optimization of agricultural planting structure in order to achieve the goal of saving irrigation water and energy and sustainable use of cultivated land in the NCP.

## 2. Materials and Methods

### 2.1. Study Area

**Figure 1:**
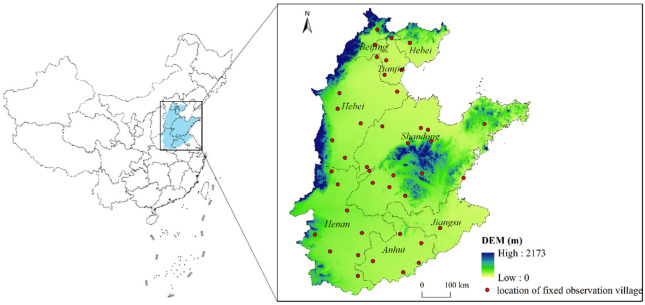
Locations of the study area and fixed observation villages.

The NCP (32°19′ N–40°18′ N 112°18′ E–120°25′ E) lies in northeastern China and includes Beijing, Tianjin, Shandong, and parts of Hebei, Jiangsu, and Anhui provinces, covering an area of approximately 4.4 × 10^5^ km^2^ (Figure 1). The average altitude is lower than 50 m; the annual average temperature and precipitation are 13 °C and 710 mm, respectively. The main soil type is fluvial soil with a deep profile and a loamy texture, which is suitable for food production. The main grain crops include winter wheat, summer maize, spring maize, and rice. Specifically, winter wheat is usually sown in early October and harvested in the following June; summer maize is usually sown in early June and harvested in late September; and spring maize is usually sown in early May and harvested in late August, while rice is also sown in early May but harvested in late September.

### 2.2. Data Source and Preprocessing

The crop harvested area maps, proportion of irrigated area, meteorological data, agricultural input data, and net primary productivity (NPP) data were mainly used in this study.

The crop harvested area maps (2000 and 2015), with a spatial resolution of 1 km × 1 km, were derived from the Institute of Remote Sensing and Digital Earth, Chinese Academy of Sciences [5]. These maps were generated by locating the harvested area of winter wheat, summer maize, spring maize, and rice onto maps of cultivated land area, mainly following the three-step method described by Monfreda et al. [28]. These maps were not only used to analyze changes in grain planting structure in the NCP, but also used to calculate the IWC and EC of four main grain crops.

The proportions of irrigated area for four main crops around the year 2000, with a spatial resolution of 5 arc seconds, were calculated using the rain-fed and irrigated areas sown to crops, obtained from the MIRCA 2000 data [29]. The details of the calculation can be found in Wang et al. [25]. These data were used to quantify the IWC of crops.

Daily meteorological data for the period of 2000–2015, including maximum and minimum temperature, precipitation, relative humidity, wind speed, and sunshine duration, were downloaded from the China Meteorological Data Sharing Service System (http://data.cma.cn/ (accessed on 1 January 2018)). They were also used to calculate the IWC of four main grain crops in the NCP.

The agricultural input data include the costs of machinery, irrigation, pesticide, and plastic film, and the consumption of nitrogen, phosphate, potash, and compound fertilizer for wheat, maize, and rice. These data were mainly collected from the rural fixed observation point system in China (2003–2015) [30]. The detailed description of the rural fixed observation point system can be found in reference [31].

The monthly NPP data for the period 2000–2015, with a spatial resolution of 1 km × 1 km, were obtained from the Resource and Environment Science and Data Center of the Chinese Academy of Sciences (https://www.resdc.cn/ (accessed on 1 July 2020)). They were used to interpolate the EC of four main grain crops onto crop harvested area maps.

### 2.3. IWC of Grain Crops at the Pixel Level

The Chinese edition of the Agro-Ecological Zones (China-AEZ) model was applied to quantify the pixel-level IWC of four main grain crops in the NCP, which was co-developed by the Institute of International Applied Systems Analysis (IIASA) and Shanghai Meteorological Bureau during the major NSFC-IIASA cooperation project: *Assessing the impact of climate change and incentive human activities on China’s agro-ecosystem and its supply potentials* (2010–2012).

The China-AEZ model can simulate the process of crop growth and is often applied for calculation of crop productivity and water consumption [10,32]. The validity of its results has been proved by many studies [33,34]. Specifically, the IWC of grain crops at the pixel level can be calculated as:

(1) $$IWC_{ij}=\sum_{t=2000}^{2015}\left(ET_{c,ij}^{t}-EP_{ij}^{t}\right)\times IRR_{ij}/16$$

where $IWC_{ij}$ refers to the annual average IWC of crop *i* in pixel *j* during 2000–2015. $ET_{c,ij}^{t}$ and $EP_{ij}^{t}$ refers to the actual water evapotranspiration and effective precipitation of crop *i* in pixel *j* in year *t* under irrigation condition, respectively. The units of the above three indicators are both mm. The specific steps of calculating *ET_c_* and *EP* by the China-AEZ model can be found in references [25,35]. $IRR_{ij}$ refers to the proportion of irrigated area for crop *i* in pixel *j.* Due to data availability, the proportions of irrigated area for crops in 2000 were applied throughout the study period 2000–2015.

It should be pointed out that the annual average IWC during 2000–2015 are calculated for four main crops, offsetting the influences of extreme weather incidents and other circumstances. Therefore, this study only concerns the impact of changes in grain planting structure on IWC in the NCP.

### 2.4. EC of Grain Crops at the Pixel Level

Energy consumed by grain crops in the NCP includes not only the fuel and electricity consumed during the sowing, irrigation, and harvesting of crops, but also the energy consumed during the production processes of fertilizer, pesticides, and plastic films. Due to data availability, EC during the transportation of agricultural products is not considered in this study. In this sense, the EC of grain crops in the NCP can be roughly divided into five aspects: mechanical energy consumption, irrigation energy consumption, fertilizer energy consumption, pesticide energy consumption, and plastic film energy consumption. The relevant formulas are as follows:

(2) $$EC_{it}=EC_{it}^{fu}+EC_{it}^{ir}+EC_{it}^{fe}+EC_{it}^{pe}+EC_{it}^{pf}$$

where $EC_{it}$ refers to the total EC of crop *i* in year *t* (MJ/ha). $EC_{it}^{fu}$, $EC_{it}^{ir}$, $EC_{it}^{fe}$, $EC_{it}^{pe}$, and $EC_{it}^{pf}$ refer to the fuel energy consumption, irrigation energy consumption, fertilizer energy consumption, pesticide energy consumption, and plastic film energy consumption of crop *i* in year *t* (MJ/ha), respectively.

(3) $$EC_{it}^{fu}=\frac{C_{it}^{fuel}+C_{it}^{machinery}\times R^{fuel}}{P_{t}^{fuel}}\times N_{fuel}$$

where $C_{it}^{fuel}$ and $C_{it}^{machinery}$ refer to the costs of fuel power and machinery for crop *i* in year *t*, respectively (RMB/ha). $R^{fuel}$ refers to the proportion of fuel cost in machinery cost, which is used to separate the cost of fuel from the cost of machinery, usually taken as 40% according to the machinery operators [36]. $P_{t}^{fuel}$ refers to the price of fuel in year *t*, and the price of diesel oil is used here because it is the main fuel used in the agricultural production process. $N_{fuel}$ refers to the EC coefficient of diesel oil [37].

(4) $$EC_{it}^{ir}=\frac{C_{it}^{irrigation}-C_{it}^{water}}{P_{t}^{electricity}}\times N_{electricity}$$

where $C_{it}^{irrigation}$ and $C_{it}^{water}$ refer to the cost of irrigation and water resource for crop *i* in year *t*, respectively (RMB/ha). Considering that irrigation cost in the NCP includes not only the electricity but also water costs, water costs should be deducted from EC calculation. $P_{t}^{electricity}$ refers to the price of electricity used for irrigation in year *t* (RMB/KW h). $N_{electricity}$ refers to the EC coefficient of electricity [37].

(5) $$EC_{it}^{fe}=\sum_{x=1}^{4}fe_{it}^{x}\times N_{x}$$

where $fe_{it}^{x}$ refers to the amount of fertilizer *x* for crop *i* in year *t* (kg/ha). *x* refers to the type of fertilizer, i.e., nitrogen, phosphorus, potassium, and compound fertilizer. $N_{x}$ refers to the EC coefficient of fertilizer *x* [38].

(6) $$EC_{it}^{pe}=pe_{it}\times N_{pe}$$

where $pe_{it}$ refers to the amount of pesticide for crop *i* in year *t* (mL/ha). $N_{pe}$ refers to the EC coefficient of pesticide [37].

(7) $$EC_{it}^{pf}=pf_{it}\times N_{af}$$

where $pf_{it}$ refers to the amount of plastic film for crop *i* in year *t* (kg/ha). $N_{af}$ refers to the EC coefficient of plastic film [37].

The ECs of four main grain crops in fixed observation villages were first calculated year by year using the above formulas. Considering that crop input is usually strongly correlated with crop growth condition, and that NPP is a commonly used proxy for crop growth condition [39,40], crop EC of fixed observation villages can be downscaled to the cropping plating area in the NCP by means of the NPP during the crop growth period. The specific formula is as follows:

(8) $$EC_{ij}^{t}=EC_{it}\times \frac{NPP_{ij}^{t}}{\mathrm{AVG}\left(NPP_{ij}^{t}\right)}$$

where $EC_{ij}^{t}$ refers to the total EC of crop *i* in pixel *j* in year *t* (MJ/ha). $NPP_{ij}^{t}$ refers to the NPP during the growth period of crop *i* in pixel *j* in year *t* (gC/ha). $\mathrm{AVG}\left(NPP_{ij}^{t}\right)$ refers to the mean NPP during the growth period of crop *i* in year *t* within a certain region. Considering the spatial distribution of fixed observation villages (Figure 1), this region is selected as municipality. For the municipalities without fixed observation villages, their crop EC is replaced by that of the fixed observation village of the nearest municipality.

Similar with the IWC dataset of grain crops at the pixel level, a spatially explicit dataset of annual average EC for four main grain crops during 2003–2015 is constructed using the year-by-year data calculated by Equation (8) for the purpose of accurately assessing the impact of changes in grain planting structure on crop EC in the NCP. All the above processes were done using ArcGIS 10.3 (Environmental Systems Research Institute, Redlands, CA, USA).

### 2.5. Coupling Index of IWC and EC of Grain Crops at the Pixel Level

Taking the idea of measuring the coupling relationship between urbanization and the environment [41], a new coupling index of IWC and EC (CI) was constructed to detect the coupling relationship of these two factors not only for four main grain crops, but also for grain production in the NCP.

(9) $$CI_{ij}=\sqrt{\left(\frac{\bar{IWC_{ij}}\times \bar{EC_{ij}}}{\bar{IWC_{ij}}+\bar{EC_{ij}}}\right)\times \left(0.5\times \left(\bar{IWC_{ij}}+\bar{EC_{ij}}\right)\right)}$$

where $CI_{ij}$ refers to the coupling index of IWC and EC for crop *i* in pixel *j*. $\bar{IWC_{ij}}$ and $\bar{EC_{ij}}$ refer to the standard scores of the IWC and EC for crop *i* in pixel *j*, and their formulas are as follows:

(10) $$\bar{IWC_{ij}}=\frac{IWC_{ij}-\min\left(IWC_{ij}\right)}{\max\left(IWC_{ij}\right)-\min\left(IWC_{ij}\right)}$$

(11) $$\bar{EC_{ij}}=\frac{EC_{ij}-min\left(EC_{ij}\right)}{\max\left(EC_{ij}\right)-min\left(EC_{ij}\right)}$$

where $IWC_{ij}$ has the same meaning as that in Equation (1), and $EC_{ij}$ refers to the annual average EC for crop *i* in pixel *j*. In this context, a higher CI value implies a higher coupling degree and higher scores of both IWC and EC. However, considering the environmental effects of IWC and EC, a high CI value also implies a high environmental burden and low sustainability in crop production.

CI for grain production in the NCP can be obtained using the following formula:

(12) $$CI_{t}=\underset{i}{\sum }\underset{j}{\sum }\left(\frac{Area_{ij}^{t}}{\sum Area_{ij}^{t}}\times CI_{ij}\right)$$

where $CI_{t}$ refers to the CI values for grain production in year *t*. $Area_{ij}^{t}$ refers to the area of crop *i* in pixel *j* in year *t* (km^2^). All these processes were also done using ArcGIS 10.3 (Environmental Systems Research Institute, Redlands, CA, USA).

## 3. Results

### 3.1. Changes in Grain Planting Structure in the NCP

**Figure 2:**
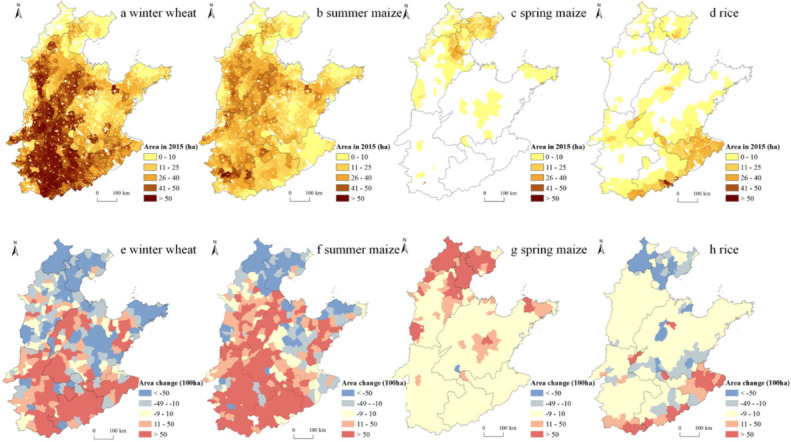
Spatial distribution of sown area at the kilometer grid scale in 2015 (**a**–**d**) and changes in sown area at the county level for four grain crops in the NCP from 2000 to 2015 (**e**–**h**).

The spatial distribution patterns of four main grain crops differed significantly in the NCP in 2015 (Figure 2a–d). Specifically, winter wheat and summer maize had roughly the same spatial distribution pattern, concentrated in southern Hebei, northwestern and southwestern Shandong, and Henan and Anhui, with less distribution in the remaining areas of the NCP. Spring maize was mainly concentrated in northern Hebei, Beijing, and Tianjin, as well as the central hills and eastern coastal areas of Shandong, while few was grown in the rest of the NCP. Rice was mainly distributed in the northern part of Hebei, Beijing, Tianjin, the southern part of the NCP, and also in southern and northwestern Shandong province, with Jiangsu being the most densely distributed.

**Figure 3:**
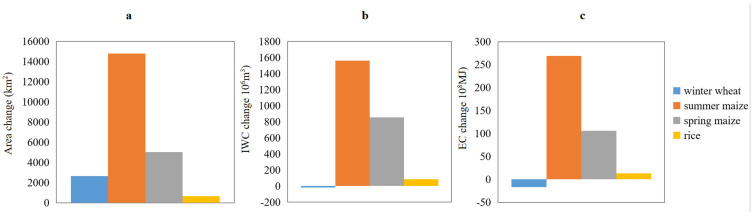
Changes in sown area (**a**), irrigation water consumption (IWC) (**b**) and energy consumption (EC) (**c**) of four grain crops in the NCP from 2000 to 2015.

From 2000 to 2015, the sown areas of four main grain crops all increased in the NCP, with winter wheat, summer maize, spring maize, and rice increased by 2630.73 km^2^, 14781.73 km^2^, 5024.33 km^2^, and 669.19 km^2^, respectively (Figure 3a). Spatially, the winter wheat sown area contracted in the northern part of the NCP, i.e., the Beijing-Tianjin-Hebei region (BTH), and expanded in the southern region, including Henan, Anhui, Jiangsu, and the northwestern and southwestern parts of Shandong (Figure 2e). Except for Beijing, Tianjin, northern Hebei, and the central hills and eastern coastal areas of Shandong, where the sown area of summer maize contracted, other regions of the NCP expanded (Figure 2f). For spring maize, the sown areas of counties where it distributed showed an increase (Figure 2g). The sown area of rice showed a pattern of contraction in the north and expansion in the south margins (Figure 2h).

### 3.2. Changes in IWC and EC for Grain Production in the NCP

**Figure 4:**
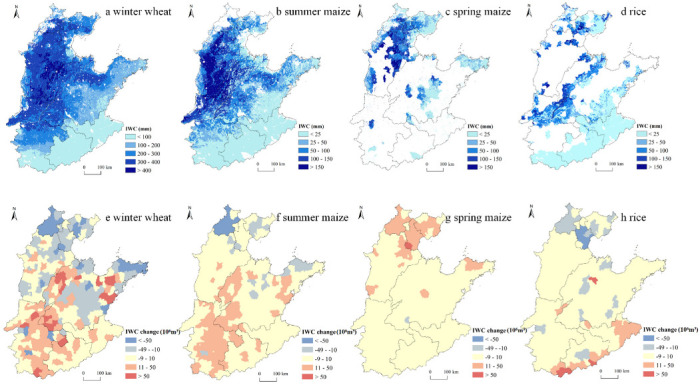
Spatial distribution of IWC in 2015 (**a**–**d**) and changes in IWC at the county level for four grain crops in the NCP from 2000 to 2015 (**e**–**h**).

**Figure 5:**
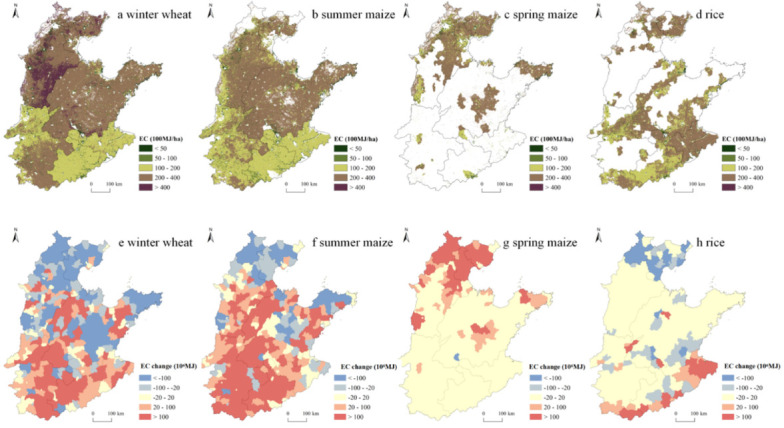
Spatial distribution of EC in 2015 (**a**–**d**) and changes in EC at the county level for four grain crops in the NCP from 2000 to 2015 (**e**–**h**).

Among the four main grain crops in the NCP, the IWC of winter wheat was the highest, with high value areas concentrated in the BTH, where the IWC of winter wheat was higher than 300 mm, while in the southern region, including Anhui and Jiangsu, the IWC of winter wheat was slightly lower, less than 100 mm (Figure 4a). The IWCs of the remaining three grain crops were within 200 mm across the NCP, also showing a pattern of more in the north and less in the south. Specifically, the IWCs of all three crops in the BTH were above 100 mm, while those in Jiangsu and Anhui were less than 25 mm (Figure 4b–d). With respect to ECs, those of winter wheat, summer maize, and spring maize all showed a pattern of large in the north and small in the south, especially in Anhui and Jiangsu (Figure 5a–c). In comparison, EC of rice was large not only in northern NCP, but also in Jiangsu, while small values were mainly concentrated in southern Henan (Figure 5d).

From 2000 to 2015, the total IWC and EC for winter wheat in the NCP decreased 19.87 × 10^6^ m^3^ and 16.78 × 10^8^ MJ, respectively (Figure 2b,c). Specifically, as the area of winter wheat increased in the south and decreased in the north, the change pattern of EC was basically consistent with that of area for this crop (Figure 5e). In comparison, counties with a decrease of IWC were more concentrated in the northern BTJ, and central and eastern coastal areas of Shandong, and counties with an increase of IWC were more concentrated in northwestern and central-eastern Shandong, and within Henan, while Jiangsu and Anhui experienced less change (Figure 4e). With respect to summer maize, the total IWC and EC in the NCP increased 1559.79 × 10^6^ m^3^ and 268.81 × 10^8^ MJ, respectively (Figure 2b,c). Specifically, counties with large decreases in EC were mainly concentrated in northern BTJ and central and eastern coastal areas of Shandong, while other counties of the NCP mainly experienced an increase in EC. In comparison, counties with large decreases in IWC for summer maize shrank to Beijing, and counties with large increases in IWC shrank to Henan and western Shandong (Figure 4f and Figure 5f). With respect to spring maize, the total IWC and EC in the NCP increased 854.17 × 10^6^ m^3^ and 105.77 × 10^8^ MJ, respectively (Figure 2b,c), and both IWC and EC increased in counties where this crop was planted (Figure 4g and Figure 5g). With respect to rice, the total IWC and EC in the NCP increased 85.51 × 10^6^ m^3^ and 13.22 × 10^8^ MJ, respectively (Figure 2b,c), and both IWC and EC decreased in counties in the BJH and increased in the southern margins of the NCP. Moreover, EC for rice also decreased in counties of southern Shandong (Figure 4h and Figure 5h).

### 3.3. Changes in the Coupling Relationship of IWC and EC for Grain Production in the NCP

**Figure 6:**
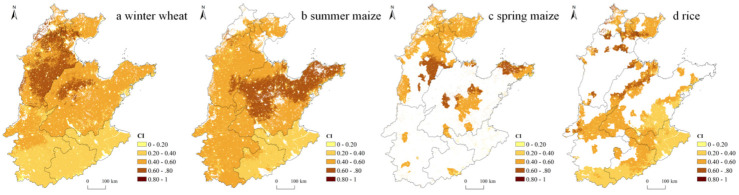
Spatial distribution of the CI for four grain crops in the NCP in 2015: (**a**) winter wheat, (**b**) summer maize, (**c**) spring wheat, (**d**) rice.

The spatial distribution patterns of CI for the four main grain crops were also different (Figure 6a–d). Specifically, the high CI values of winter wheat were mainly concentrated in Hebei and northwestern Shandong, while the CI values were lower in Anhui, Jiangsu, and southern Henan, implying that winter wheat production in Hebei was highly irrigation water- and energy-intensive and placed a heavier burden on the environment, while the opposite was true in the south. With respect to summer maize and spring maize, their high CI values both were concentrated in southeastern Hebei and northern Shandong, indicating that the production of maize in these areas was also highly irrigation water- and energy-intensive and relatively less sustainable. Compared with other crops, the areas with high CI values for rice were scattered in northern Hebei and northwestern Shandong, and the CI values were lower in the rest of the NCP, indicating a low degree of water-energy coupling.

**Figure 7:**
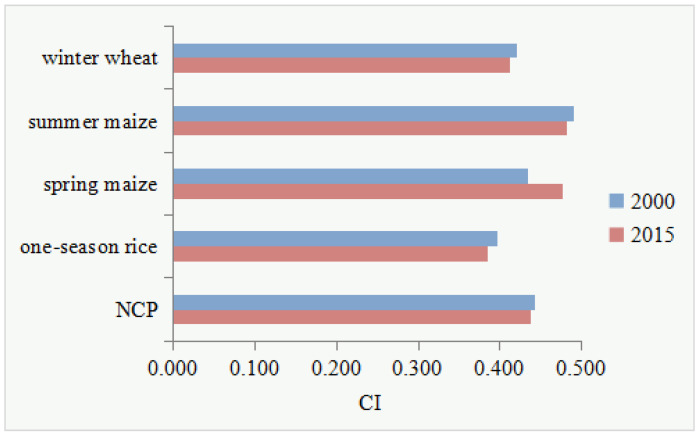
Changes in CI for four grain crops in the NCP from 2000 to 2015.

With the spatial changes in the sown area of the four main grain crops, their overall coupling indices also changed (Figure 7). Specifically, the CIs of winter wheat, summer maize and rice decreased from 2000 to 2015, from 0.420, 0.490, and 0.397 in 2000 to 0.413, 0.482, and 0.385 in 2015, respectively. This indicated that the high water and energy consumption in the production process of these three crops was weakened, the burden on the environment was correspondingly reduced, and sustainability improved. Only the CI of spring maize increased, from 0.434 in 2000 to 0.478 in 2015, indicating its placement to high irrigation water- and energy-consuming areas and thus increased pressure on the environment. Overall, CI values for gain production in the NCP decreased from 0.442 in 2000 to 0.438 in 2015, implying that the grain crop distribution has been optimized toward a less water- and energy-intensive and more sustainable layout.

## 4. Discussion

In this study, we constructed a new CI index, i.e., the water-energy coupling index for grain production. This index can make an integrative assessment of the IWC and EC during crop production, which can not only realize the analysis of the spatial-temporal change of the water-energy coupling degree in the production process of a single crop, but also systematically evaluate the change of the water-energy coupling relationship under the background of regional crop planting structure change in the NCP. Furthermore, it can provide a reference for the in-depth analysis of the FEW nexus. The results revealed that from 2000 to 2015, CI values for grain production in the NCP showed a decreasing trend, from 0.442 to 0.438, where the CI values of winter wheat, summer maize, and rice all decreased, and only that of spring maize increased. Therefore, against the background of the “expand in the south and shrink in the north” in the sown areas of winter wheat, summer maize, and rice, the grain production in the NCP shifted to the southern region with lower water and energy consumption, and the coupling relationship between high IWC and high EC for food production has changed to the coupling relationship between low IWC and low EC, exerting less pressure on the environment of the NCP.

Using the three metrics including the IWC and EC and their coupling index CI, this study can help detect the disadvantage and excess of irrigation water and energy consumption and measure the coordination degree of two elements in main grain production. In this context, this study has the potential to serve as a guidance of better spatial optimal allocation of crop production in the NCP and is also meaningful in providing support for the policy formulation of the reduction of water and energy uses in the agricultural sector. Specifically, we suggest continuing to optimize the layout of grain crops towards an environmental-friendly way. Relevant measures include consolidating the achievements of the cultivated land rehabilitation policy in the Heilonggang region of Hebei province, and appropriately expanding the pilot areas of this policy to continue reducing the sown area of winter wheat in northern NCP where the production of winter wheat is high irrigation-water intensive and high energy intensive [42,43]. At the same time, in Henan, Jiangsu, and Anhui, the sown area of winter wheat-summer maize and winter wheat-rice double cropping system should be increased through moderate subsides to ensure a stable grain supply of the NCP. In addition, measures such as improvement of drop-tolerant varieties of crops, advanced experiences of field management including no-tillage, and soil testing and formula fertilization should be implemented in the NCP to further reduce the intensity of IWC and EC of grain crops [22,44].

Finally, we would like to acknowledge the uncertainties of this study. Considering that autumn crops such as spring maize, summer maize, and rice may have the homospectral phenomenon of foreign bodies in the growing season, it is difficult to accurately identify them through remote sensing images [45]. Therefore, the crop harvested area maps, i.e., localization of the harvested area of crops onto maps of cultivated land area, were adopted, which may not be completely consistent with the actual distributions of crops in the NCP. In addition, the EC of four main crops was calculated using data from the rural fixed observation point system in China, which is the most detailed data including the input and output data during crop production we can obtain at present [31]. However, it should be noted that the fixed observation villages within the NCP are still limited. In the future, large-scale household survey can be conducted to obtain more crop input-output data, so as to construct more accurate spatially explicit data of crop energy consumption.

## 5. Conclusions

As one of the major grain producing areas in China, the contradiction between grain production and water resources in the NCP is prominent, and therefore most studies have focused on the water consumption of its grain production. In this paper, we analyzed not only the changes of IWC during the change of grain planting structure in the NCP, but also the changes of its EC and the changes of the coupling relationship between IWC and EC. The results showed that winter wheat and summer maize were mainly distributed in the eastern and southern areas in the NCP and spring maize was mainly concentrated in the BTH, while rice was mainly located in the northern BTH and in Anhui and Jiangsu. The IWC and EC for each type of crop all roughly decreased from north to south in the NCP. From 2000 to 2015, the sown areas of winter wheat, summer maize, and rice all showed a trend of increase in the south and decrease in the north. Only the area of spring maize increased in most counties where it was planted. Crop planting structure changes resulted in decreases of IWC and EC for winter wheat but increases of IWC and EC for the other three grain crops. Specifically, the IWC and EC of winter wheat and rice decreased mainly in the BJH region and increased in the southern parts of the NCP. In comparison, except for northern BTJ and central and eastern coastal areas of Shandong, most of the other regions experienced an increase in IWC and EC for summer maize. For spring wheat, both IWC and EC increased in its growing areas. From 2000 to 2015, the CI values of winter wheat, summer maize, and rice all decreased in the NCP, while the CI values of spring maize increased, and the total CI values for grain production in the NCP also showed a decreasing trend. In this context, grain crop distribution has been optimized toward a less water- and energy-intensive and more sustainable layout in the NCP.

This study can not only reveal the change of the water-energy coupling relationship in the context of regional crop planting structure change and contribute to the case studies of the FEW nexus but also can provide policy suggestions for regional crop planting structure optimization and conservation of water and energy resources.
